# Generalized recovery algorithm for 3D super-resolution microscopy using rotating point spread functions

**DOI:** 10.1038/srep30826

**Published:** 2016-08-04

**Authors:** Bo Shuang, Wenxiao Wang, Hao Shen, Lawrence J. Tauzin, Charlotte Flatebo, Jianbo Chen, Nicholas A. Moringo, Logan D. C. Bishop, Kevin F. Kelly, Christy F. Landes

**Affiliations:** 1 Department of Chemistry, Rice University, Houston, TX 77251, USA; 2 Department of Electrical and Computer Engineering, Rice University, Houston, TX 77251, USA

## Abstract

Super-resolution microscopy with phase masks is a promising technique for 3D imaging and tracking. Due to the complexity of the resultant point spread functions, generalized recovery algorithms are still missing. We introduce a 3D super-resolution recovery algorithm that works for a variety of phase masks generating 3D point spread functions. A fast deconvolution process generates initial guesses, which are further refined by least squares fitting. Overfitting is suppressed using a machine learning determined threshold. Preliminary results on experimental data show that our algorithm can be used to super-localize 3D adsorption events within a porous polymer film and is useful for evaluating potential phase masks. Finally, we demonstrate that parallel computation on graphics processing units can reduce the processing time required for 3D recovery. Simulations reveal that, through desktop parallelization, the ultimate limit of real-time processing is possible. Our program is the first open source recovery program for generalized 3D recovery using rotating point spread functions.

Single-molecule and super-resolution imaging^1^,^2^,^3^,^4^,^5^ have changed our understanding of biological processes^6^,^7^, dynamics at interfaces^8^,^9^,^10^,^11^,^12^, and functions of catalysts at the single molecule level^13^,^14^,^15^. Molecular motors are now known to move hand-over-hand^5^,^16^. Stochastic ligand clusters complicate chromatographic protein separations^17^,^18^. Cheaper and higher resolution gene mapping is made accessible^19^,^20^. The inner functions of live bacteria are unveiled^21^,^22^.

Underpinning each new piece of super-resolved knowledge are similarly revolutionary advancements in image analysis algorithms. For example, using 2D Gaussian fitting instead of the center of mass is critical to achieve nanometer scale resolution^23^. In order to speed up the analysis process, fast fitting algorithms that address both mathematical analysis^24^,^25^ and computational resources^26^ have been proposed. Algorithms focused on high-density imaging such as DAOSTORM^27^, compressed sensing interpolation^28^ and FALCON^29^, have inspired the development of super-resolution imaging with better time resolution. Today, we have a variety of sophisticated algorithms for recovery under different measurement conditions in 2D super-resolution imaging.

More recently, 3D super-resolution imaging is finding increased applications. Cells and organelles all have 3D structures^30^,^31^, and many biological processes^32^ and separation processes are 3D processes^17^. It is urgent to develop 3D super-resolution techniques with time and space resolution comparable to 2D techniques. One approach is to scan over different z positions and record multiple 2D images, with Ober’s group demonstrating simultaneous multiple detection planes to image 3D motion in living cells^33^. One advantage of such hardware-based methods is that the generated image can be analyzed by 2D processing algorithms.

Another popular method is to encode the phase information (which is related to the z position of the emitter) in the intensity distribution by using a cylindrical lens (astigmatism)^30^ or phase mask^34^,^35^,^36^ in the detection path so that 3D information is recorded in a single 2D image. Different phase masks generate different 3D point spread functions (PSFs), as shown in Supplementary Fig. S1 and references^35^,^37^,^38^,^39^. The advantages of astigmatism-based methods are that they are cheaper and have lower hardware requirements making them accessible to a broader group of researchers. However, data analysis becomes a challenge because, for most phase-based measurements, the PSF cannot be simply approximated and fit by simple equations like the 2D Gaussian function^40^,^41^. Algorithms such as 3D DAOSTORM can analyze astigmatism-based 3D images^42^. 3D FALCON^43^ can be used to analyze astigmatic images, biplane images, as well as hybrid images that combine astigmatic and biplane methods^43^. However, PSFs recorded in these types of 3D microscopies can be well-fit by Gaussians or elliptical Gaussians, which is usually not possible for phase mask engineered PSFs. Easy-DHPSF is a useful algorithm to analyze 3D single-particle tracking data using a double-helix phase mask^41^, but it requires the recorded PSFs to have no overlap, which, as we discuss below, is a serious challenge for most 3D recovery algorithms. Barsic *et al*. introduced an algorithm to analyze such 3D imaging data^44^, but the algorithm is not open source and thus not generalizable. Despite the promise of phase mask-based 3D imaging and isolated successful implementation, there is still a need for algorithms to analyze broad types of experimental images, as well as to provide reliable test-data for comparing performance between different phase masks.

A major challenge in phase mask based 3D super-resolution imaging is recovering accurate 3D localizations from a range of analyte densities^45^,^46^,^47^. As mentioned earlier, when using a phase mask, 3D information is projected onto 2D images with overlapped PSFs that are individually more complicated than a simple Gaussian. Often, the resulting overlapping PSFs can prevent accurate localization unless the distribution of the excited emitters is sparse in the space domain^28^,^29^. Extracting accurate 3D localizations with higher emitter densities is preferred, but this experimental requirement increases the challenges of subsequent image recovery. Therefore, the processing efficiency of 3D super-resolution recovery algorithms is important in practice.

In order to address the importance of accuracy, precision, and processing speed, we introduce a 3D super-resolution recovery algorithm for emitters imaged with arbitrary 3D phase masks that generate rotating PSFs. We use an alternating direction method of multipliers (ADMM)^48^,^49^,^50^,^51^ based algorithm to deconvolute the sample positions from the 3D measurement, which records a single 2D image with encoded 3D information. We further improve the resolution by using a Taylor expansion to calculate the 1^st^ order corrections between these grids^29^ using least squares fitting. ADMM is a powerful and efficient algorithm for convex optimization^49^,^52^. Moreover, we apply a threshold generated by machine learning (ML) to reject false positive identifications. Thresholding based on machine learning makes use of features from the data that are difficult to capture based on human observations^53^,^54^,^55^,^56^,^57^,^58^. In addition, we show how the recovery algorithm can be implemented both on a central processing unit (CPU) and a graphics processing unit (GPU). By using an affordable GPU, it is possible to increase processing speeds by an order of magnitude. Further estimation shows that by using a GPU array it would be possible to reach real-time data analysis of even dense phase mask data. To our knowledge, our algorithm will serve as the first open source algorithm for 3D recovery using phase mask imaging. Finally, as a proof-of-concept, we demonstrate that our algorithm can be used to localize single molecules within the 3D structure of a porous polystyrene film.

## Results and Discussions

**Figure 1:**
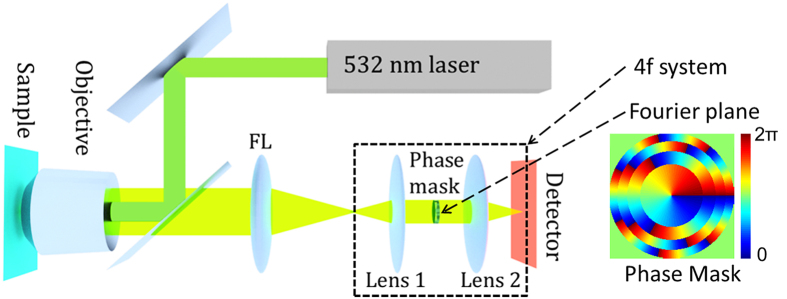
Schematic of our 3D super-resolution microscope using a phase mask. FL is the focal lens. For a typical wide field microscope, the detector is placed at the focal point after FL. Lens 1 and Lens 2 are two identical lenses forming a 4f system. The phase mask is mounted in the Fourier plane, which is the center plane between Lens 1 and Lens 2. The detector is placed after the 4f system. The phase mask is made of transparent materials with different thicknesses generating different phase delays. The simulated phase mask pattern shown approximates the double helix phase mask reported elsewhere^61^, and the commercially available phase mask (Double Helix LLC) used in the experimental portions of the current work.

One common 3D super-resolution approach is to incorporate a 4f system into the detection path of a traditional wide field microscope (Fig. 1). This 4f system is composed of two identical lenses (L1 and L2) separated by twice the focal distance and a phase mask mounted in the focal plane^35^,^59^,^60^ between the two lenses. This plane is called the Fourier plane, which is the ideal location to manipulate the phase pattern in the detection path. The 4f system does not change the magnification. In x and y dimensions, the magnification is:


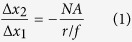


In the z dimension, the magnification is:


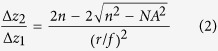


where Δ*x*_1_ (Δ*x*_2_) and Δ*z*_1_ (Δ*z*_2_) are the displacements in the x-y plane and in the z direction on the sample side (detector side) respectively, *NA* is the numerical aperture of the objective, *n* is the refraction index of the working medium for the objective, *r* is the effective beam radius, which should equal to the radius of the phase mask, and *f* is the focal distance of Lens 1 and Lens 2, as shown in Fig. 1.

For a rotating PSF, the orientation (or shape) of the PSF as a response to different z positions is 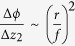, where Δ*ϕ* represents the orientation (or shape) change. Combining with equation (2), we have: 
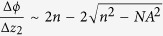
. This means the orientation (or shape) response of a phase mask is only related to the objective. However, based on equation (1), the magnification of the PSF in x and y are inversely proportional to the ratio of beam radius and focal distance 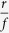.

**Figure 2:**
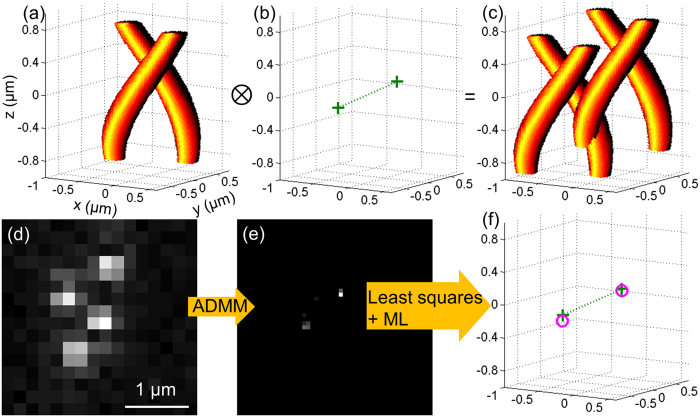
Illustration of the 3D imaging process with a double-helix phase mask (a–c) and the super-resolution recovery procedures (d–f). (**a**) A double-helix 3D PSF is generated by a phase mask. Using this 3D PSF, the depth information of an emitter is indicated by the relative orientation of the two lobes in the x-y plane. (**b**) Two simulated emitters (dark green crosses) separated in 3D space. The dashed line is used to guide the eye. (**c**) The full 3D image space that results from a convolution of the double-helix PSF with the 3D positions of the two emitters. (**d**) A simulated CCD image that would occur from placing a photodetector at the focal plane (z = 0 μm) of the image space described by the convolution of the double-helix PSF and the two emitters. (**e**) The recovered positions of the emitters in front view (x-y plane) on the grids using ADMM algorithm. (**f**) The final recovered positions using least squares fitting and machine learning to avoid overfitting (magenta circles) compared with the ground truth (dark green crosses).

The imaging process of this 3D microscope can be modeled as the convolution of a 3D PSF (such as the double helix PSF as shown in Fig. 2a) with emitters positioned in 3D (Fig. 2b), which generates overlapping 3D PSFs (Fig. 2c). The detector only records the 2D image at z = 0 plane, as shown in Fig. 2d. This incomplete sampling of the imaging space causes difficulty in later deconvolution for super-resolution recovery.

Recovering a super-resolution 3D image reduces to a convex optimization problem. We assume the emitter distribution (like Fig. 2b) is approximated by a 3D matrix *x*, and the 3D PSF (like Fig. 2a) is represented by the 3D matrix *A*. We use a 2D matrix *y* to store our measured image (like Fig. 2d). To find *x*, we need to solve the optimization problem:


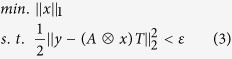


in which ⊗ means convolution, *T* is a 1D vector, and *ε* is the tolerance of the noise. The minimization is over *x*. The only non-zero element in *T* corresponds to the z = 0 image from the convoluted 3D matrix. The 3D matrix *A* ⊗ *x* needs to be reshaped into a 2D matrix. The two horizontal dimensions become the first dimension and the z dimension is the second dimension. Multiply this reshaped 2D matrix with the 1D vector *T* selects the re-constructed image at z = 0, which can be used to compare with the measured image *y*. In single molecule experiments, the excited emitters in every recorded image are sparse^28^. Recent developments in compressive sensing and sparse sampling have demonstrated that using the L_1_ norm can recover sparse signals exactly^28^,^62^,^63^. The constraint in the second line ensures agreement between the measured image and the recovered image. However, directly solving this optimization problem requires a large amount of memory and computation resources, making this 3D optimization problem infeasible for most personal computers.

Recently published ADMM based deconvolution algorithms^50^,^51^ break down the optimization problem into multiple sub-problems to accelerate the computation and reduce the memory requirement by using circular convolution. Based on variable splitting and Lagrange multipliers, the solution of the optimization problem (eq. 3) can be found by solving:


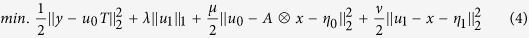


Here we use *u*_0_ (*u*_1_) to replace *A*⊗*x* (*x*) in the first (second) term and force them to be the same in the third (fourth) penalty term; and *η*_0_, *η*_1_, *μ* and *ν* are related to Lagrange multipliers (for a complete understanding of Lagrange multipliers and ADMM, please review reference^49^). We use *μ* = 1 and *ν* = 20 based on ref. 50 for the best performance. The new optimization problem can be solved iteratively by updating one unknown at a time. In iteration *k* + 1, these unknowns can be updated in this way:


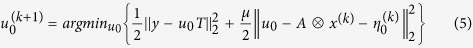



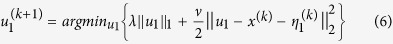



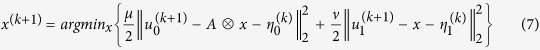



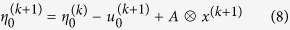



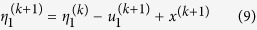


where *argmin* means argument of the minimum, which means finding the value of the variable that minimize the expression. Equation (5), (6) and (7) can be solved explicitly without any iteration, which is the major reason for the state-of-the-art speed of this algorithm. Moreover, *A* ⊗ *x* now can be calculated more efficiently using a fast Fourier transform (FFT), and variables like *x*, *η*_0_ and *η*_1_ can be updated in the Fourier domain without an inverse Fourier transform. This further reduces the number of required operations. We use 1000 iterations for all the analysis in this work. The sparsity of the solution is guaranteed by soft thresholding (eq. 6), which is equivalent to the L_1_ norm^50^,^51^. This has been shown in ref. 64. An ADMM algorithm handles much larger images at a time compared to directly solving the convex optimization problem (eq. 3)^28^,^44^. However, solutions resulting from ADMM deconvolution are on discrete grids and are vulnerable to overfitting. As shown in Fig. 2e, more than two emitters (corresponding to the bright pixels) are identified, meaning there are overfittings after ADMM deconvolution.

We further improve the resolution via least squares fitting and suppress overfitting using a machine learning determined threshold (Fig. 2f). In the deconvolution algorithm, the 3D PSF is approximated as a discrete 3D matrix. By adding the 1^st^ order Taylor expansion in the *x, y* and *z* directions, we can approximate the 3D PSF in continuous space^29^ and refine the positions of the emitters by solving this least squares problem:


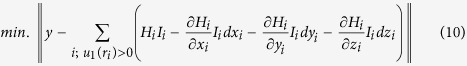


where *H*_*i*_, 
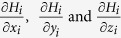
 are the corresponding 2D PSFs and the 1^st^ order Taylor expansions in *x, y*, and *z* in the imaging plane for emitter *i*; *I*_*i*_ is the intensity of emitter *i*; *dx*_*i*_, *dy*_*i*_, *dz*_*i*_ are 1^st^ order differentials in *x*, *y* and *z* directions.

**Figure 3:**
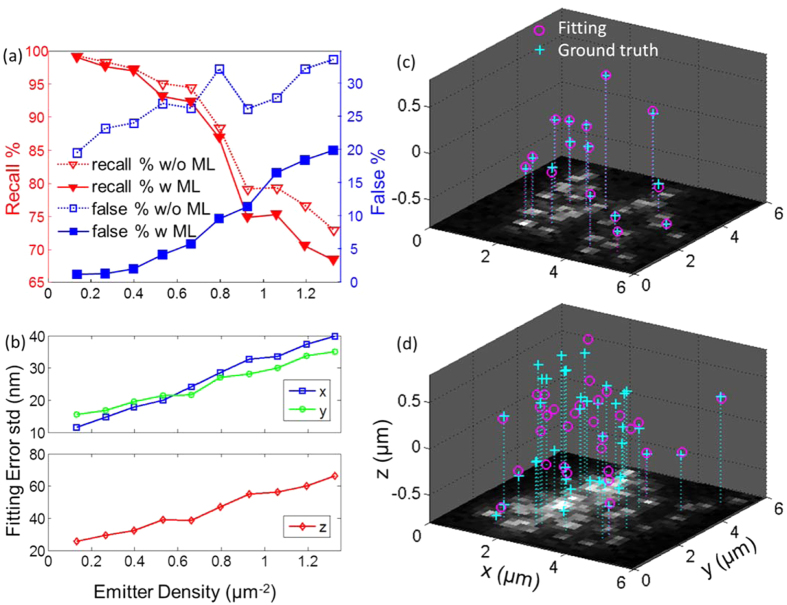
Performance of the algorithm. (**a**) Recall rate (recall %, left axis, in red color) and false positive rate (false %, right axis, in blue color) with or without the ML step. The standard deviation of each point is shown in Supplementary Fig. S3. (**b**) The standard deviation of the fitting error distribution in *x* (blue square), *y* (green circle) and *z* coordinates (red diamond). (**c**) Example recovery result of an image with 15 emitters (emitter density = 0.4 μm^−2^) in a 3D plot. The recovered vs. simulated true positions are indicated in magenta circles and cyan crosses, respectively. The simulated measured image is shown in the bottom of the 3D space. All the emitters are located with no overfitting. (**d**) Example recovery result of an image with 40 emitters (emitter density = 1.06 μm^−2^) in a 3D plot. There are many incorrect identifications, which can lead to misrepresentations about the sample.

Least squares fitting alone cannot distinguish true positive emitters and false positive emitters. Research in super-resolution recovery frequently focuses on recall rates (the number of identified true positive emitters over the number of all true emitters), and focuses less on the false positive rate (the number of identified false positive emitters over the number of all the emitters identified by the algorithm). However, the false positive rate is equally, if not more, important. A lower recall rate is a matter of measuring time but a higher false positive rate potentially distorts the true structure. Usually researchers use pre-selected thresholds (such as 5% of the highest intensity) to remove false positives. We instead use labeled data and ML to find out a more objective threshold via multiple parameters (see SI for details). As shown in Fig. 3a, using this training data determined threshold, the recall rate decreases by a small amount, but the false positive rate decreases significantly.

Based on our simulations (simulation details are explained in the SI), the optimal emitter density in 3D imaging can be determined. As shown in Fig. 3a,b, as the emitter density increases, the recall rate decreases and false positive rate increases, and the fitting error in every dimension increases. The fitting error as a function of emitter density increases in a linear trend, but the recall rate shows a gradual decrease when the emitter density is greater than 0.7 μm^−2^. The false positive rate for emitter density larger than 0.8 μm^−2^ is larger than 10%, meaning a larger possibility of identifying misleading structures. As illustrated in Fig. 3c, at lower emitter densities, we can recover almost all of the emitters with no overfitting. At higher densities (Fig. 3d), false positive emitters are more likely to be identified. Based on this simulation, we suggest keeping emitter density smaller than 0.7 μm^−2^ in 3D imaging measurements. Under these guidelines, simulations have shown we can correctly recover 3D structures with high labeling density with ~10 nm resolutions (Supplementary Fig. S4). Our choice of emitter densities range within commonly used in the field of super-resolution microscopy using a visible light laser^65^ and widely discussed in other works^28^,^44^. These general guidelines are consistent for a range of different phase masks other than the double-helix (Supplementary Fig. S2). This simulation test on a different phase mask also proves the performance of the machine learning determined threshold and further demonstrates that our algorithm can be used to evaluate the performance of new phase mask designs. This program can be downloaded from our website: http://lrg.rice.edu/Content.aspx?id=96.

Despite our efficient and generalizable algorithm, more than one hour is required to analyze a 512 × 512 image on a typical personal computer with a standard CPU (Intel i7-4770, 3.40 GHz). To optimize the processing time, we need to parallelize the computation. Such parallelization is easily possible using a GPU. If an algorithm can break down the problem into multiple independent floating point operations, parallel computation on a GPU has been shown to accelerate the processing by 10–100 times compared to computation on a high-end CPU^26^. A GPU conducts thousands to millions of independent floating point operations simultaneously. Image processing is an ideal application for GPUs. Each pixel of the image can be assigned to a thread and different threads can perform similar operations simultaneously.

ADMM based image deconvolution is accelerated by an order of magnitude through computation on a GPU (Table 1). Operations in ADMM algorithms are primarily related to 3D FFT and matrix-matrix element wise operations, in which the 3D FFT is the most time consuming part. The parallel computing platform created by NVIDIA, which is called compute unified device architecture (CUDA), provides a library for FFT on GPUs (http://www.nvidia.com/). We make use of this FFT library for fast convolution and deconvolution computations and distribute all other element wise operations to millions of threads. Parallel computation speeds up our algorithm by 10 times using a GeForce GTX 645 GPU (576 CUDA cores, 1GB global memory), as shown in Supplementary Fig. S5 and Table 1. The limits of achievable acceleration are the number of CUDA cores, which decides the number of threads being processed at a time, and the amount of global memory, which limits the amount of data being processed at a time. Usually, the number of CUDA cores is the only limiting factor. If we use a high-end GPU, such as the NVIDIA Tesla K80 with 4992 CUDA cores, the speed of our computation can increase by an additional factor of ten (Table 1). For a typical single-molecule measurement, we can record 1000 images in 30 s, and the data size is approximately 1 GB. The NVIDIA Tesla K80 has 24 GB of memory with 480 GB/s bandwidth, so data transfer time is instantaneous and we won’t face the limitation of the memory. Therefore, data analysis speed increases linearly as the number of Tesla K80 to be used in parallel. With the current development of parallel computation, one can envision that in the not-so-distance future, real-time analysis will be possible and affordable with the extension to parallel GPU processing.

**Figure 4:**
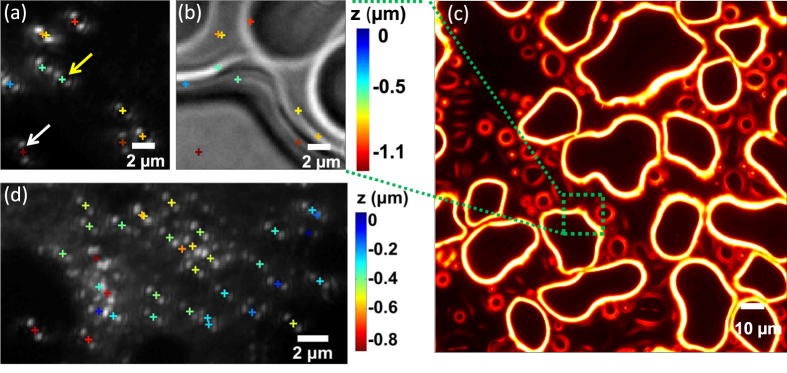
3D super-localization of 40 nm orange fluorescent bead adsorption onto porous polystyrene films. Details about sample preparation can be found in the SI. (**a**) 3D localization of fluorescent beads on a porous polystyrene film with (**b**) correlated bright-field image of the corresponding area. The cross markers in (**a,b**) indicate the 2D positions of identified emitters. The color of each marker indicates the relative z position of each emitter, as shown in the corresponding color bar. The arrows highlight two emitters at different depths corresponding to positions within a pore and on the edge of a pore. (**c**) A dark-field image of the polystyrene film structure. The region studied in (**a,b**) is labelled in the green box. (**d**) 3D localization is demonstrated on another polystyrene film with a higher density of beads. The phase mask used to collect the experimental data was purchased from Double Helix LLC.

As an example, we show the application of our recovery algorithm to nanoscale 3D structures in porous polystyrene films (Fig. 4). Engineered polymer films are used in chemical and biological separations, and understanding analyte/film interactions has been of recent interest^18^. Correlating the connection between the 3D morphology of the polymer films and the separation efficiency might provide a means to produce films with improved separation performance^8^,^11^,^17^,^66^,^67^. Previous studies focused on dynamic interactions between analytes and clustered-charge ligands imbedded in the support film, but also suggested that nanoscale heterogeneities in film structure are also important^17^,^44^,^63^,^65^. The proof-of-concept analysis shown in Fig. 4 suggests that our algorithm can be combined with more complicated analytes and samples to directly relate nanoscale 3D film structure and dynamic interactions between the analyte and the film.

We prepared a porous polystyrene film and drop casted orange fluorescent beads onto the film (see SI for details about sample preparation and data acquisition). As shown in Fig. 4a, the local depth of each bead was extracted. Figure 4b and c are the bright-field (Fig. 4b) and dark-field (Fig. 4c) images of the same area. As would be expected with a porous film, the distribution of super-localized bead depths suggests that some of the beads are within pores (e.g. highlighted by the white arrow) whereas some are on the film surface (e.g. highlighted by the yellow arrow). The depth localizations of beads agree well with the underlying surface morphology of the porous film. Moreover, we also used our algorithm to analyze another porous film with a higher density of beads (Fig. 4d), strongly supporting that our algorithm can be used to perform 3D super-localization and super-resolution even when the complex PSFs generated by phase masks are overlapped.

## Conclusion

We have demonstrated via simulation that our new algorithm can recover a 3D super-resolution image measured by a 3D microscope using phase masks in the Fourier plane. In the development of our algorithm, we leveraged state-of-the-art techniques in signal processing and optimization including ADMM and machine learning, as well as advanced computation resources to achieve the best possible algorithm performance and with computations completed in a few seconds via GPU processing. Our algorithm could play an important role in future data processing tasks including performance testing for new phase mask development. Our current algorithm still requires a good match between the experimental PSF and the simulated PSF. Motion blur in 3D single molecule tracking and complicated background in imaging will affect the performance of our algorithm. For our future work, we will further improve our algorithm for more complicated experimental conditions.

## Additional Information

**How to cite this article**: Shuang, B. *et al*. Generalized recovery algorithm for 3D super-resolution microscopy using rotating point spread functions. *Sci. Rep.* **6**, 30826; doi: 10.1038/srep30826 (2016).

## Supplementary Material

Supplementary Information

## Figures and Tables

**Table 1:** Computational speed comparison between CPU and GPU.

Image size (pixels)	CPU (Intel i7-4770)	GPU (GeForce GTX 645, 576 CUDA cores)	GPU (Tesla K80, 4992 CUDA cores)
	Matlab R2013a	CUDA C	CUDA C
8 × 8	12.6 s	0.85 s	<0.1 s
56 × 56	105 s	8.5 s	~1 s
120 × 120	439 s	33.1 s	~4 s
192 × 192	1054 s	98.3 s	~11 s
