# Supplementary material for: Generalized recovery algorithm for 3D super-resolution microscopy using rotating point spread functions
Source: Sci Rep. 2016 Aug 4;6:30826. doi: 10.1038/srep30826 (PMC4973222; doi:10.1038/srep30826)
Supplement: Supplementary Information [file srep30826-s1.pdf]

SI:

Generalized recovery algorithm for 3D super-resolution microscopy using rotating point spread functions

Bo Shuang[1], *Wenxiao Wang*[2], *Hao Shen*[1], *Lawrence J. Tauzin*[1], *Charlotte Flatebo*[1], *Jianbo Chen*[2], *Nicholas A. Moringo*[1], *Logan D.C. Bishop*[1], *Kevin F. Kelly*[2], *Christy F. Landes*[1,2,*]

[1] Department of Chemistry, Rice University, Houston, TX 77251

[2] Department of Electrical and Computer Engineering, Rice University, Houston, TX 77251

AUTHOR INFORMATION

Corresponding Author

*Phone: 713-348-4232. E-mail: cflandes@rice.edu

**Supplementary Note**	Supplementary text about simulations and experiments
**Supplementary Figure S1**	Different phase mask designs
**Supplementary Figure S2**	Performance tests of dumbbell PSF and tri-lobe PSF
**Supplementary Figure S3**	Recall rate and false positive rate in Fig. 3a with standard deviation plotted as error bars
**Supplementary Figure S4**	Simulation tests using 3D structures with high labeling densities
**Supplementary Figure S5**	Processing time of ADMM deconvolution on a CPU compared to processing time on a GPU for images of different sizes

Supplementary Note

Simulation details

Simulation data are generated following the 3D convolution process discussed in Fig. 2a-c. First, a number of emitters are randomly located in a 3D image space (like Fig. 2b). The x and y dimensions of the sample space are approximately 6 μm (32 image pixels in both x and y dimensions) and the z dimension is approximately 1.6 μm (corresponding to 21 layers in the simulated PSF matrix). The number of photons emitted by each emitter follows a Poisson distribution with mean of 2000 photons. The noise for each pixel follows a Poisson distribution with a mean of 20 photons. For each case of different emitter densities, approximately 1000 emitters in total are simulated and recovered using our algorithm. Mapping the recovered emitters and the simulated true positions is an assignment problem. The best match will have the minimum total Euclidean distance between the recovered emitters and true emitters[1]. We set a 1 pixel threshold detecting incorrect assignments. A recovered emitter having no true emitter within a 1 pixel distance is recorded as a false positive. Similarly, a true emitter lacking a recovered emitter within a 1 pixel distance is recorded as a false negative. Only matched emitters are used to calculate the fitting errors, as shown in Fig. 3b and Supplementary Fig. S2b and d. Training data for the machine learning algorithm consisted of 100 simulated images with a particle density of 0.5 μm^{-2}. The data used in Fig. 3 was employed as testing data for the algorithm. The effective testing data is the same data used in Fig. 3 as explained in detail above.

Machine learning thresholding

Overfitting is a severe problem in super-resolution image analysis, especially when PSFs overlap (Fig. 3c and d). Therefore, in many algorithms, the designer will get rid of dim particles, as these particles are more likely to be false positive identifications. However, this threshold is ill-defined and can be difficult to set. Some algorithms use 5% of the largest intensity[2], but it depends on the situation; a 5% threshold may not be large enough to get rid of all the false positive results.

In this work, we applied machine learning to define a threshold with simulated images rather than establish a universal, pre-set intensity threshold. This thresholding is a vector operator that is not only applied to signal intensities, but also considers emitter position updates during consecutive iteration. Machine learning is powerful in complicated classification problems. In our case, we want to separate the false positive identifications from the true identifications and remove all false positives. We first analyzed 100 simulated images (the density is approximately 0.5 μm^{-2}) and labeled all true identifications and false positives. We applied the least squares fitting algorithm five times to update the position for each identified emitter. The intensity at each step is divided by the averaged intensity, and the change in position in each update is used to update the position. Labeling identified emitters is similar to particle tracking. As such, we used our previous particle tracking algorithm to identify emitters, allowing a search radius of 1 pixel. Using this labeled training data, we trained our simple logistic regression model:

$$y = \frac{1}{1 + \exp(-\Theta X)}$$

where Θ is the vector operator, X contains the relative intensity and position updates of all the identified emitters, and y indicates if a emitter is true ($y > 0.5$) or false ($y \leq 0.5$), which is the commonly accepted assignment in logistic regression[3]. After the training, Θ can be used to indicate if an identified emitter is true or not. Even though the training is only conducted on one type of phase mask (phase mask 2 in Supplementary Fig. S1), the machine learning prediction works well on the other phase masks (phase mask 3 and 4, see results in Supplementary Fig. S2a and c) and experimental data (Fig. 4).

Experimental porous polystyrene films and data collection

1. Preparation and characterization of porous polystyrene film

The porous polystyrene film was prepared via the breath figure method[4]. A pre-cleaned coverslip was placed in an environment of water vapor saturated air, and 100 μL 1 wt% polystyrene (Sigma-Aldrich, Mw = 36k) in toluene solution was drop casted onto the coverslip. While the toluene evaporated, water droplets condensed on the surface of polystyrene, forming micron-sized pores. The final porous film was obtained once the toluene was completely evaporated. The entire film formation process took around 5 minutes to finish. The resulting polystyrene films contained pores with various sizes, as demonstrated by dark field microscopy (Fig. 4d in the main text).

2. Loading 40 nm orange fluorescent beads to the porous film

The carboxylate-modified microspheres (0.04 μm) were purchased from Invitrogen (stock number F-8792), which have a fluorescent emission maximum around 560 nm. The stock solution was diluted by 10^6 times in HEPES buffer (pH = 7.3, GIBCO). Diluted bead solution (100 μL) was drop cast onto the porous polystyrene film and dried in air to generate the final sample for fluorescence microscope measurements. The polystyrene beads adsorbed to the polymer film surface and pore edges due to the intrinsic hydrophobicity of polystrene. Performing 3D super-localization analysis of the adsorbed beads reveals the underlying 3D surface morphology of the film.

3. DH PSF 3D microscopy

The 3D detection data in Fig. 4 was collected by a home-built DH PSF 3D microscope. A 532 nm solid state laser (Coherent, Compass 315M – 100SL) is used to excite the fluorescent emitters. The laser beam is focused at the center of a 1.45 numerical aperture, 100 × oil-immersion objective (Carl-Zeiss, alpha Plan-Fluar) in epi-fluorescence excitation mode. The fluorescent signal is separated by a dichroic mirror (Chroma, z532/633rpc) and then transferred into the 4f system after the tube lens with two 4f lenses (Thorlabs, Matched Achromatic Doublet Pairs) and transmitted through a DH phase mask (Double Helix LLC, Double helix phase mask). Final data signal is recorded with a sCMOS camera (Hamamatsu, ORCA-Flash 4.0).

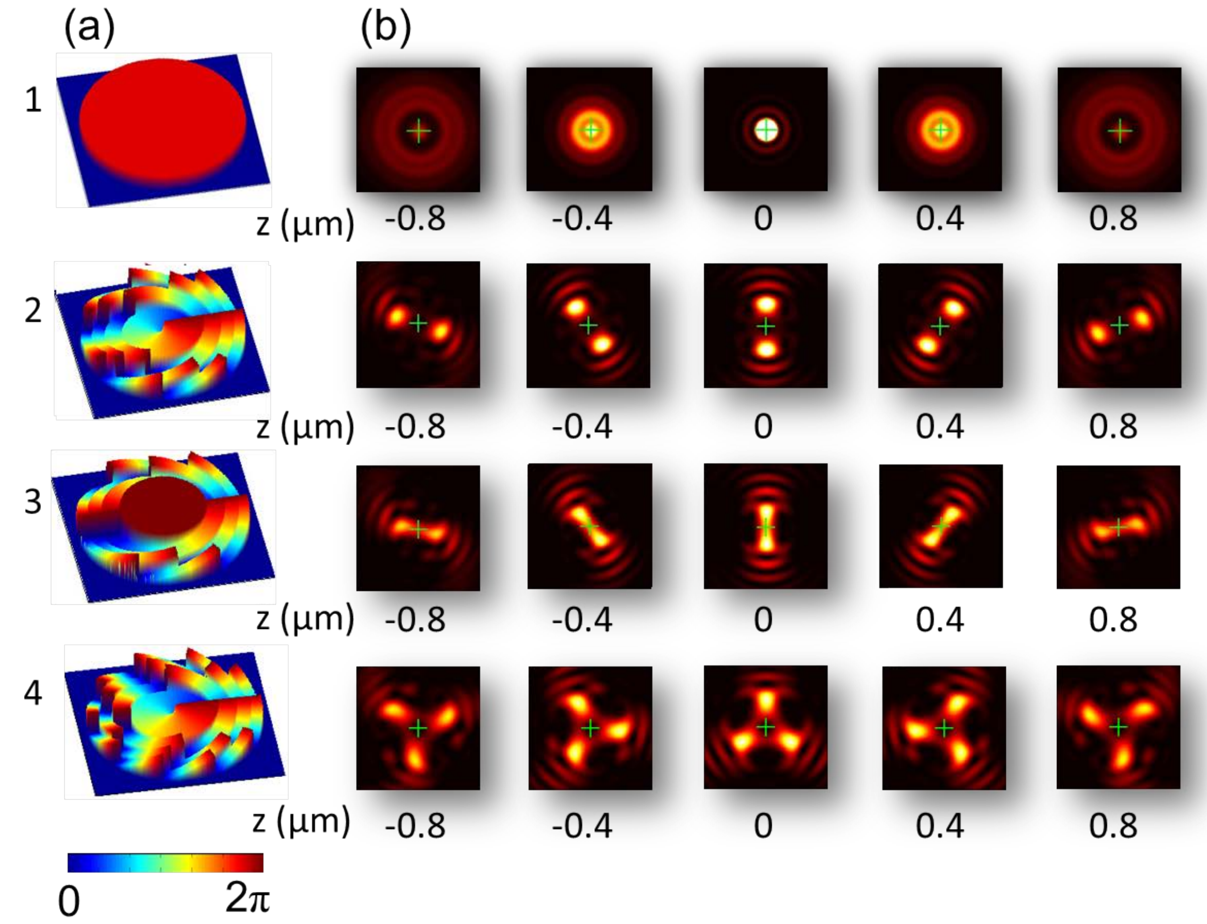

Supplementary Figure S1. Phase masks (a) and the corresponding PSF patterns for emitters at different z positions (b). Different colors in (a) represent different phase delays. For a transparent phase mask, the phase delay is proportional to the thickness of the material[5]. Uniformly distributed phase masks and samples without a phase mask generate standard Gaussian PSFs (row 1). A Gaussian PSF provides a high peak intensity when the emitters are at the focal plane, but the peak intensity drops quickly as the emitters drift away from the focal plane. The symmetry in peak intensity does not allow for a Gaussian PSF to indicate the direction of the drift. A double-helix phase mask generates a rotating PSF as a function of focal depth (row 2). The decrease of the peak intensity as a function of the z position is much slower than a Gaussian PSF, making this rotating PSF a better option for 3D imaging. Because we do not have access to the actual double-helix phase mask pattern, we use a similar pattern to mimic the double-helix PSF in this work[6]. We change the phase mask pattern to generate alternative rotating PSFs[7], referred to as a dumbbell PSF (row 3) and tri-lobe PSF (row 4).

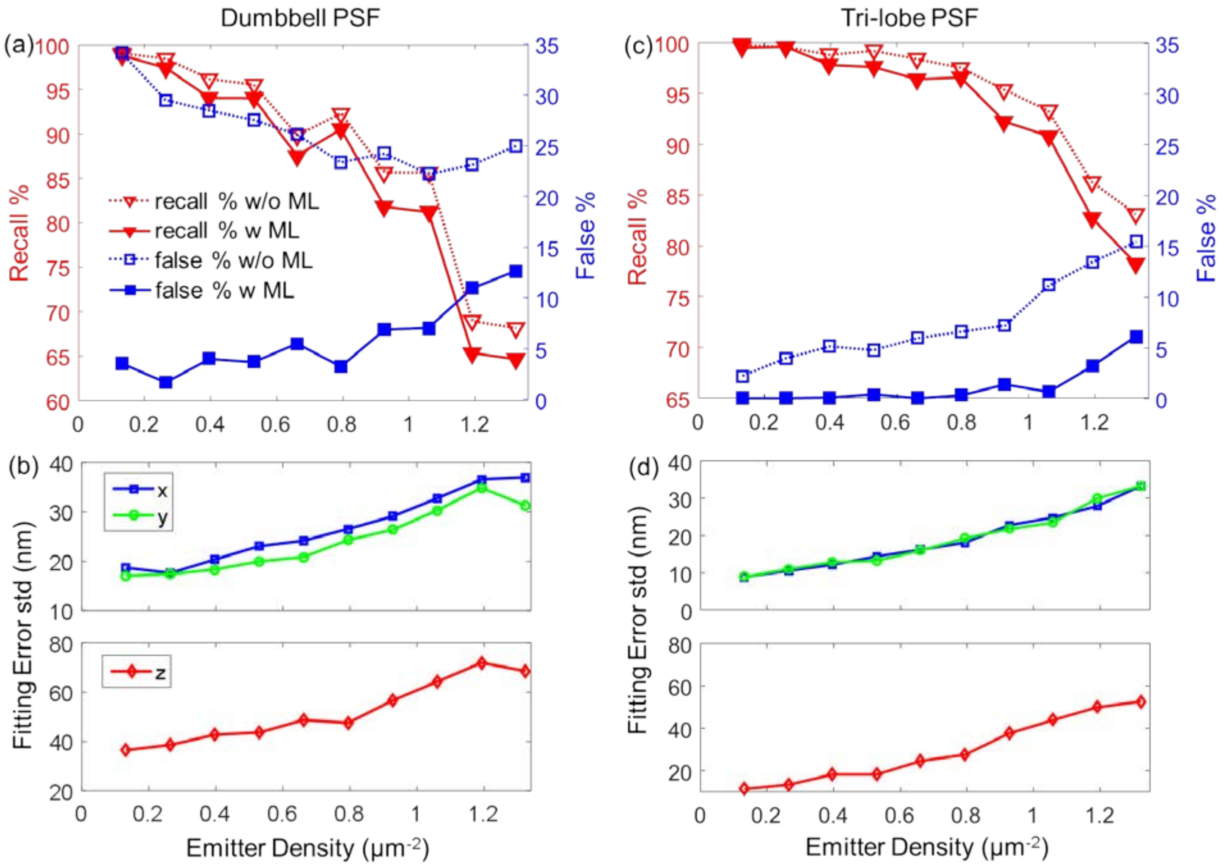

Supplementary Figure S2. Performance tests using the dumbbell PSF (a, b) and tri-lobe PSF (c, d). (a, c) Recall rate and precision rate with or without the ML step. (b, d) Standard deviation of the fitting error distribution in x (blue square), y (green circle) and z coordinates (red diamond). Overall, the performance of the two PSFs show a similar trend compared to the double-helix PSF.

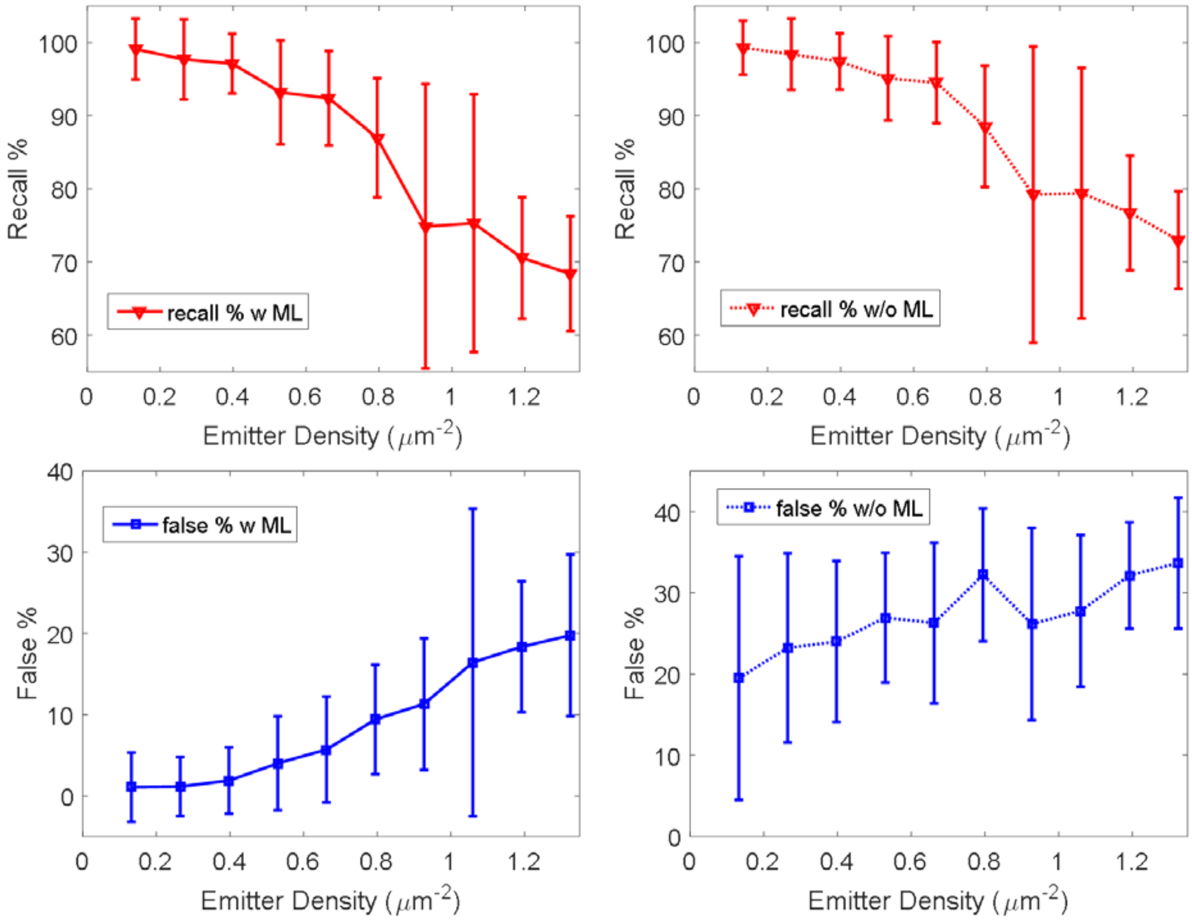

Supplementary Figure S3. Recall rate and false positive rate in Fig. 3a with standard deviation plotted as error bars.

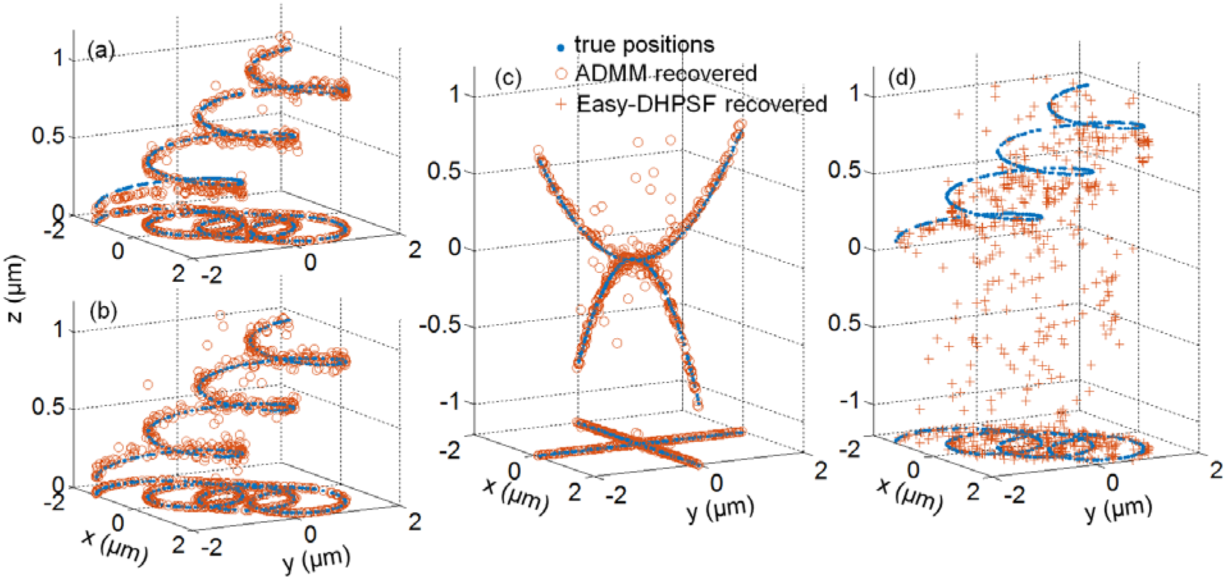

Supplementary Figure S4. Simulations of imaging 3D structures with high labeling densities and overlapping PSFs. (a) Simulated 3D circular structure and the recovered positions using our method. SNR=30. (b) The same test but SNR = 7. (c) Simulated 3D saddle shape structure and the recovered positions using our method with SNR = 30. (d) Simulated 3D circular structure and the recovered positions using Easy-DHPSF[8]. SNR = 30. For all the tests, 100 frames are simulated and analyzed for each test. The particle density is approximately 0.3 μm^{-2} in each frame.

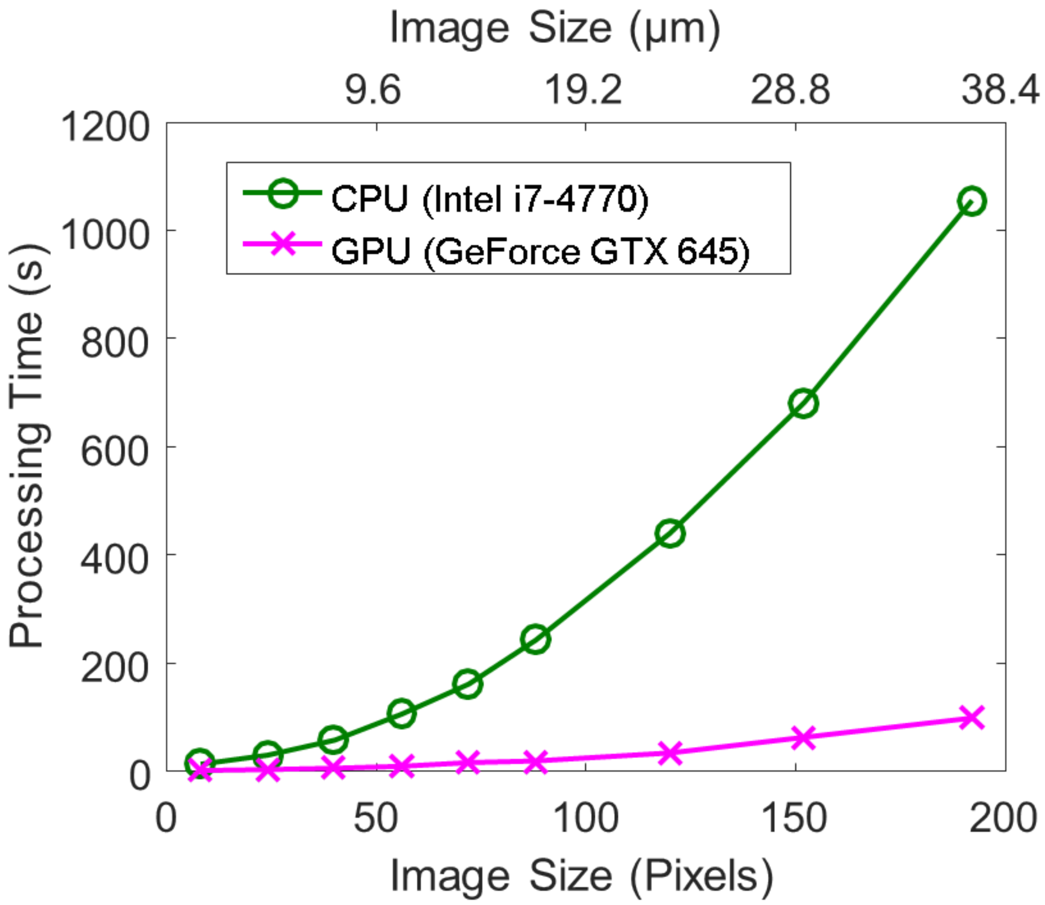

Supplementary Figure S5. Processing time of ADMM deconvolution on a CPU compared to processing time on a GPU for images of different sizes.

References

1. Shuang, B., Chen, J., Kisley, L. & Landes, C.F. Troika of single particle tracking programing: SNR enhancement, particle identification, and mapping. *Phys. Chem. Chem. Phys.* **16**, 624-634 (2014).
2. Min, J. et al. FALCON: fast and unbiased reconstruction of high-density super-resolution microscopy data. *Sci. Rep.* **4**, 4577 (2014).
3. Guzella, T.S. & Caminhas, W.M. A review of machine learning approaches to Spam filtering. *Expert. Syst. Appl.* **36**, 10206-10222 (2009).
4. Zhang, A., Bai, H. & Li, L. Breath Figure: A Nature-Inspired Preparation Method for Ordered Porous Films. *Chemical Reviews* **115**, 9801-9868 (2015).
5. Grover, G., Quirin, S., Fiedler, C. & Piestun, R. Photon efficient double-helix PSF microscopy with application to 3D photo-activation localization imaging. *Biomedical optics express* **2**, 3010-3020 (2011).
6. Pavani, S.R. et al. Three-dimensional, single-molecule fluorescence imaging beyond the diffraction limit by using a double-helix point spread function. *Proc Natl Acad Sci U S A* **106**, 2995-2999 (2009).
7. Prasad, S. Rotating point spread function via pupil-phase engineering. *Opt. Lett.* **38**, 585-587 (2013).
8. Lew, M.D., von Diezmann, A.R.S. & Moerner, W.E. Easy-DHPSF open-source software for three-dimensional localization of single molecules with precision beyond the optical diffraction limit. *Protoc. Exch.* **2013**, 026 (2013).
